# Retraction: ZFP36 binds with PRC1 to inhibit tumor growth and increase 5-Fu chemosensitivity of hepatocellular carcinoma

**DOI:** 10.3389/fmolb.2025.1561889

**Published:** 2025-01-23

**Authors:** Frontiers Editorial Office

The journal retracts the 14 July 2020 article cited above.

Following publication, concerns were raised regarding the integrity of the images in the published figures. The authors failed to provide a satisfactory explanation during the investigation, which was conducted in accordance with Frontiers’ policies.

This retraction was approved by the Chief Executive Editor of Frontiers. The authors received a communication regarding the retraction and do not agree to this retraction. This communication has been recorded by the publisher.

